# Novel Insight into Pickering Emulsion and Colloidal Particle Network Construction of Basil Extract for Enhancing Antioxidant and UV-B-Induced Antiaging Activities

**DOI:** 10.1021/acsomega.2c07657

**Published:** 2023-04-26

**Authors:** Rizzqi Septiprajaamalia Rosdianto, Nurul Zakiyah, Rafiqah Nur Viviani, Fortunata Saesarria Deisberanda, Tantri Liris Nareswari, Satrialdi, Irda Fidrianny, Damar Rastri Adhika, William Xaveriano Waresindo, Unang Supratman, Tri Suciati

**Affiliations:** † Department of Pharmaceutics, School of Pharmacy, Bandung Institute of Technology, Jl. Ganesha 10, Bandung, 40132, Jawa Barat, Indonesia; ‡ Department of Pharmacy, Faculty of Science, Sumatera Institute of Technology, South Lampung 35365, Indonesia; § Department of Pharmaceutical Biology, School of Pharmacy, Bandung Institute of Technology, Jl. Ganesha 10, Bandung 40132, Jawa Barat, Indonesia; ∥ Department of Engineering Physics, Faculty of Industrial Engineering, Bandung Institute of Technology, Jl. Ganesha 10, Bandung 40132, Jawa Barat, Indonesia; ⊥ Doctoral Program of Physics, Faculty of Mathematics and Natural Sciences, Institut Teknologi Bandung, Jl. Ganesha 10, Bandung 40132, Jawa Barat, Indonesia; # Department of Chemistry, Faculty of Mathematics and Natural Sciences, Padjajaran University, Jatinangor, 45363 Sumedang, West Java, Indonesia

## Abstract

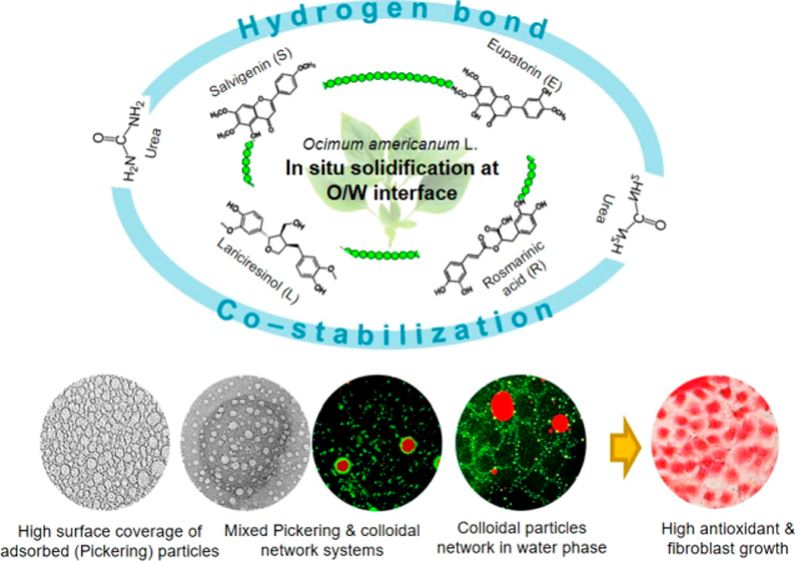

We developed a facile preparation method of oil-in-water (O/W) Pickering emulsion in an emollient formulation using basil extract (*Ocimum americanum* L.) as a solid particle stabilizer by fine-tuning the concentration and mixing steps of common cosmetic formulas, such as humectants (hexylene glycol and glycerol), surfactant (Tween 20), and moisturizer (urea). The hydrophobicity of the main phenolic compounds of basil extract (BE), namely, salvigenin, eupatorin, rosmarinic acid, and lariciresinol, supported high interfacial coverage to prevent coalescence of globules. Meanwhile, the presence of carboxyl and hydroxyl groups of these compounds provides active sites for stabilizing the emulsion using urea through the formation of hydrogen bonds. Addition of humectants directed the in situ synthesis of colloidal particles during emulsification. In addition, the presence of Tween 20 can simultaneously reduce the surface tension of the oil but tends to inhibit the adsorption of solid particles at high concentrations, which otherwise formed colloidal particles in water. The level of urea and Tween 20 determined the stabilization system of the O/W emulsion, whether interfacial solid adsorption (Pickering emulsion, PE) or colloidal network (CN). Variation of the partition coefficient of the phenolic compounds present in basil extract facilitated the formation of a mixed PE and CN system with better stability. The addition of excess urea induced interfacial solid particle detachment, which caused the oil droplet enlargement. The choice of stabilization system determined the control of antioxidant activity, diffusion through lipid membranes, and cellular antiaging effects in UV-B-irradiated fibroblasts. Particle sizes of less than 200 nm were found in both stabilization systems, which is beneficial for maximizing their effects. In conclusion, this study provides a technological platform to realize the demand for natural dermal cosmetic and pharmaceutical products with strong antiaging effects.

## 1 Introduction

Aging is associated with morphological changes and is characterized by loss of skin elasticity, wrinkles, irregular pigmentation, dryness, and roughness of the skin.^1,2^ Solar ultraviolet exposure, particularly UV-B, is the main cause of skin damage by the initiation of reactive oxygen species (ROS). UV-B light absorbed mainly in the epidermis induces the formation of ROS and transcription factors such as AP-1 and NF-kB. These factors impair collagen synthesis and lead to overproduction of matrix metalloproteinases (MMPs).^3^ Increased levels of MMP-1 break down collagen fibrils, thereby promoting the appearance of wrinkles. UV-B light also damages elastin fibers and, together with reduced collagen production, causes skin to sag. Keratinization becomes abnormal and, together with reduced keratin and ceramide, causes dry skin. Damaged skin then causes more ROS formation in the skin, which results in the imperfections of collagen and finally makes the skin rougher.^2^

Most antiaging formulations require components such as oils, humectants, and moisturizers to soothe dry skin and normalize epidermal keratinization for skin barrier repair.^4^ Sunflower oil (SFO) with a high content of linoleic acid and phenolic compounds can inhibit the chronic inflammatory response of aged keratinocytes, which reduces epidermal growth and promotes infection.^5^ It has been shown to maintain SC integrity and enhance skin hydration through peroxisome proliferator-activated receptor α agonist (PPAR-α) that increase keratinocyte proliferation and lipid synthesis.^5^ Moisturizing ingredients also help restore skin condition by forming an occlusive film on the skin surface, which controls the rate of water evaporation from the skin and by transporting hygroscopic substances capable of binding and retaining water into the SC.^6^ The uses of glycerol and urea as potent endogenous humectants and moisturizers^7,8^ are important components for reducing the severity of dry skin due to skin aging. Glycerol has been used as a major humectant in cosmetic products because it can spread easily on the skin and give a silky, soft, and nongreasy sensation favored by end consumers. The diverse effects of glycerol on the epidermis include increasing hydration barrier function and mechanical properties, inhibiting lipid phase transition, protecting against irritating stimuli, enhancing desmosomal degradation, and accelerating the wound-healing process.^8^

Plant extracts are sometimes also added to antiaging formulations as ROS scavengers. Compounds isolated from natural products are preferred as there is a strong market trend toward formulating green and eco-friendly products. Basil extract (BE) is one of the many bioactive compounds worldwide, which has been studied for its antioxidant, antiaging, anti-inflammatory, and anti-fungal properties.^9^ The antioxidant effect correlates strongly with the high content of phenolic compounds in basil, such as rosmarinic, chicoric, caffeic, and caftaric acids.^10,11^ Moreover, like other natural products, basil extract does not exhibit the side effects that are often shown by synthetic ingredients.^10^ Therefore, basil extract enriched in emollient formulation acts synergistically to restore skin barrier repair to relieve skin aging symptoms such as dryness, wrinkles, roughness, and decreased elasticity.^5,12^

With various components added in antiaging formulas, including oil and water phases, the preparations are usually emulsions, such as lotions and creams.^13^ Emulsions are thermodynamically unstable systems; therefore, the use of stabilizers for long-term stability is necessary.^14^ Currently, most emulsions have been stabilized by low-molecular-weight surfactants. However, the list of surfactants that can be used for cosmetic formulation is limited and may cause side effects such as irritation.^15,16^ Since new and less hazardous stabilization approaches have been developed, emulsion stabilized by solid particles or the so-called Pickering emulsion (PE) has received increasing interest in topical formulation.^17,18^

Compared with ordinary emulsions stabilized by synthetic low-molecular-weight surfactants, Pickering emulsion avoids the use of hazardous surfactants and shows increased stability, making it a promising new targeting system in cosmetic products. While the effect of the Pickering emulsion system on droplet size and emulsion stability in cosmetics has been extensive, most of the studies were conducted with the addition of noncosmetics excipients such as chitosan, starch, zein, soy protein, or whey protein.^19,20^ Only a few studies have used common excipients in the antiaging formula as Pickering emulsion stabilizers. Surprisingly, excipients from the antiaging formula can be used as self-Pickering emulsion, one of which is phenolic compounds. The potential of phenolics for Pickering emulsion stabilizers lies between its amphiphilic properties, where the ring structures will be adsorbed to interfacial oil globules and hydroxyl groups will form hydrogen bonds with water.^21^

This study aims to develop a preparation process for the O/W emulsification of SFO stabilized by a solid particle of basil extract. In phenolic compounds, the hydroxyl groups tend to form hydrogen bonds with water, so they are not adsorbed on the oil globules that stabilize the emulsion when the oil phase is added. The principle of emulsification in our study is the reduction of these hydrogen bonds, so that the phenolics can move at the oil–water interface to stabilize the globules. We use several strategies to suppress these hydrogen bonds, namely, increasing the temperature and adding humectants. Glycerol, hexylene glycol, and prevalent humectants used in cosmetic products were added to the formulation to lead phenolic solidification surrounding the oil globules. We also observed the stability of Pickering emulsion against the addition of urea and Tween 20 at various concentrations. To the best of our knowledge, this is the first paper to describe the stabilization of Pickering emulsion using basil extract rich in phenolic compounds in oil-in-water emollient formulation as a solution for green formulation and versatile technology for cosmetic antiaging applications.

## 2 Research Method

### 2.1 Materials

Basil leaves of *Ocimum americanum* L. were collected from Manoko farm, Lembang, Indonesia. Determination was done by the School of Life Sciences and Technology, Bandung Institute of Technology, Indonesia. The chemical ingredients used in the emulsion formula were of cosmetic grade, namely, sunflower oil (SFO) was purchased from Darjeeling, India; hexylene glycol from Solvay, Brazil; Tween 20 and glycerol from PT. Brataco, Indonesia. Ethanol food grade, methanol, urea, hydrochloric acid (HCl), isopropyl alcohol, chloroform, sulfuric acid (H_2_SO_4_), and sodium hydroxide (NaOH) were purchased from Merck, Germany. 2,2-Diphenyl-1-picrylhydrazyl (DPPH), Folin–Ciocalteau reagent, sodium carbonate (Na_2_CO_3_), citric acid, ascorbic acid, gallic acid, picric acid, quercetin, ferric chloride (FeCl_3_), Dragendorff’s reagent, Nile blue, direct red, phosphate-buffered saline (PBS), and Prestoblue reagent were purchased from Sigma-Aldrich, USA. Fetal bovine serum (FBS) was purchased from Thermo Fisher Scientific, USA. Throughout the study, deionized water was used.

### 2.2 Preparation of Basil Extract

The preparation of basil extract was carried out according to the previous study with slight modifications.^22^ Initially, 300 g of fresh basil leaves was washed and finely chopped into small pieces and then dissolved in 700 mL of solvent (70% ethanol–water contained in 1% citric acid). The mixture was then sonicated using an ultrasonic bath at a frequency of 40 kHz and a temperature range of 27–35 °C for 6 × 20 min with an interval of 20 min; then, the liquid extract was filtered. The clear solution was concentrated with a rotary evaporator (Buchi R-215, Switzerland) at 55–60 °C and 80 rpm for ethanol evaporation. The concentrate was then lyophilized with a freeze-drying apparatus (Buchi L-300, Switzerland) until the dry extract was obtained and then stored at a cold temperature (1–4 °C) for further use. The yield of the extract was calculated using the following equation
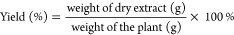
1

### 2.3 Phytochemical Screening

Ethanolic extracts of basil leaves were tested for the presence of phenolics, tannins, flavonoids, saponins, alkaloids, triterpenoids, and steroids. The tests were performed according to previous research^23−25^ with slight modifications. For the phenolic presence test, 100 mg of extract was stirred with 2 mL of distilled water and filtered. Then, a few drops of 1% FeCl_3_ were added. Black or blue-green coloration showed the presence of phenolics. Furthermore, the tannin test was carried out in the same procedure as the phenolic test but the concentration of FeCl_3_ used was 5%. Black or blue-green color or precipitate indicates the presence of tannins. Test for flavonoids was determined using three methods. The first method was the Shinoda test; 1 mL of ethanol and 3 drops of concentrated HCl were added to 0.5 mL of diluted extract in isopropyl alcohol. Then, pieces of metallic magnesium were added. The second was using 2 N H_2_SO_4_; a few drops of 2 N H_2_SO_4_ were added to 1 mL of diluted extract in isopropyl alcohol. The third was using 10% NaOH; 3 drops of 10% NaOH were added to 1 mL of diluted extract in isopropyl alcohol. The formation of a yellow-red coloration indicated the presence of flavonoids.

The test for saponin, about 100 mg of extract, was shaken with 2 mL of distilled water in a test tube. The formation of foam in the upper part of the test tube indicated the presence of saponins. Meanwhile, the presence of alkaloids was determined using the Dragendorff test. About 15 mg of extract was stirred with 6 mL of 1% HCl in a water bath for 5 min and filtered. Then, 1 mL of Dragendorff’s reagent (potassium bismuth iodide solution) was added. An orange-red precipitate showed the presence of alkaloids.

Triterpenoids and steroids were determined using the Liebermann–Burchard test. About 100 mg of extract was shaken with 2 mL of chloroform in a test tube. 2 drops of acetic anhydride were added and boiled in a water bath and rapidly cooled in iced water; then, 2 drops of concentrated H_2_SO_4_ were added alongside the test tube. The formation of a red color indicated the presence of triterpenoids, while the appearance of a bluish-green color indicated the presence of steroids.

### 2.4 Characterization of Basil Extract

The extraction result performed using 70% ethanol with ultrasonication produced a multicomponent. Therefore, fractionation of the extract was carried out before analysis using liquid chromatography and tandem mass spectrometry (LC-MS/MS). Fractionation was carried out using the gravity column chromatography (GCC) method with specifications: column height 19 cm; column diameter 2.2 cm; silica gel column G60, 19 cm × 2.2 cm (Merck Number 7734 (230 mesh), as much as 40 g; silica gel G60 7733 impregnated. The eluent used was isocratic with the composition of ethyl acetate: methanol (95:5) with the addition of a small amount of acetic acid.

A total of 16 samples were collected and analyzed using thin-layer chromatography (aluminum sheet 20 cm × 20 cm coated with silica gel 60 F254) to detect the presence of phytocompounds and then observed under UV 254 nm and UV 366 nm. Fractions 6 and 7 showed the most dominant spots; then, thin-layer chromatography (TLC) analysis was carried out to ensure that spots from fractions 6–7 were the phenolic compounds to be analyzed. Then, the analysis continued with LC-MS/MS.

A Quadrupole and Tof (Q-Tof) MS Xevo LC-MS/MS was used to identify the 6–7 fraction phenolic compounds. The ion source used was electrospray ionization (ESI) with an microchannel plate (MCP) detector in the range *m*/*z* data of 100 and 1000. The mobile phase was a gradient composition of acetonitrile and 0.01% formic acid at a flow rate of 1 mL/min. Samples were injected with several gradients, namely, 0.01% formic acid/acetonitrile of 95:5% for 3 min; 5:95% for 50 min; and 95:5% for 60 min. The conditions of the Q-TOF-MS/MS device used were capillary voltage, 3.00 kV; cone sampling voltage, 40 V; extraction cone voltage, 4 V; the temperature used when the sample was injected was 120 °C; desolvation, 250 °C; the gas flow used for the sample is a gas cone 0 L/hour; gas desolvation 800 L/hour.

### 2.5 Mixing Step Analysis and Colloidal Extract Formation

The miscibility of humectants, namely, hexylene glycol (H) and glycerol (G) with sunflower oil (SFO), was analyzed preconditioning of the extract that supported in situ colloidal formation upon contact with oil during emulsification. Several liquid mixtures were prepared such as four mixtures described here (abbreviated as BEH, BEG, BEHG, and BEH-GW), which were prepared according to the following procedure. BEH: 200 mg of dry extract was mixed with 2 mL of distilled water; then, 150 mg of hexylene glycol was added. The mixture was stirred and heated to 80 °C until perfectly mixed. BEG; 200 mg of dry extract was mixed with 2 mL of distilled water; then, 150 mg of heated glycerol (80 °C) was added. The mixture was stirred and heated to 80 °C until perfectly mixed. BEHG; 200 mg of dry extract was mixed with 2 mL of distilled water; then, 150 mg of hexylene glycol was added. The mixture was stirred and heated to 80 °C, and then 300 mg of glycerol was added, kept stirring and heated until perfectly mixed. BEH-GW; 200 mg of dry extract was mixed with 2 mL of distilled water, and then 150 mg of hexylene glycol was added. The mixture was stirred and heated to 80 °C; then, 300 mg of glycerol was added in 7.6 mL of water, kept stirring, and heated until perfectly mixed.

### 2.6 Preparation of O/W Pickering Emulsion

Preparation of the formula started with dissolving urea in 3 mL of distilled water. The dry extract was mixed with 2 mL of distilled water and then added to a half amount of hexylene glycol. The mixture was stirred and heated to 80 °C. The preparation process involved heating to 80 °C of the oil phase containing SFO, Tween 20, another half of hexylene glycol, and the water phase containing distilled water and glycerol. When the temperature reached 80 °C, the extract mixture was added to the oil phase and quickly added to the water phase. The mixture was then stirred with a digital homogenizer (Ultra Turrax T18-IKA, China) at 9000 rpm for 3 min; then, the urea solution was added and restirred for 1 min. The emulsion obtained was incubated for about 24 h at room temperature to reduce the amount of foam and was ready for characterization (Table 1).

**Table 1:** Pickering Emulsion Formula

composition	amount (%)
basil extract	2
glycerol	3
hexylene glycol	3
SFO	3
Tween 20	0, 0.2, 0.4, 0.6, 0.8, 1
urea	0, 0.5, 1, 1.5, 2
water	add 10 g

### 2.7 Surface Property Measurement of Formulation Components

Measurement of liquid surface properties, namely, surface tension and interfacial tension, was carried out to analyze its hydrophobicity and hydrophilicity as well as miscibility in the emulsification process. It was determined using a digital tensiometer (TD1 Lauda Scientific, Germany) at room temperature. Surface tension values obtained from the apparatus were uncorrected. Therefore, the correction factor (*f*) must be calculated according to the equation
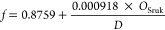
2where *O*_Sruk_ is the uncorrected surface tension (mN/m), and *D* is the specific gravity of samples (g/cm^3^). After multiplying the uncorrected surface tension value (obtained from the apparatus) by the calculated correction factor (*f*), the absolute surface tension value in mN/m was obtained.

### 2.8 Physicochemical Characterizations of Colloidal Extracts

Colloidal extracts were made to observe which components could support or prevent the formation of Pickering emulsion. The characterizations included surface tension, interfacial tension, particle size, conductivity, and pH. Surface and interfacial tension were measured using a digital tensiometer (TD1 Lauda Scientific, Germany) at room temperature. Procedures using equal methods are described in Section 2.6. The droplet size was measured using a Particle Size Analyzer Delsa Nano C (Beckman Coulter, USA). All samples were diluted 100× with distilled water before being measured at room temperature (25 °C). The conductivity values were measured using a Metrohm 712 digital conductivity meter equipped with Pt/Pt black electrodes, immediately after the preparation of samples. The measurements were conducted with gentle stirring to avoid creaming.^26,27^ The pH values were measured using a digital pH meter (Mettler Toledo SevenEasy S20, Switzerland) at room temperature.

### 2.9 Physicochemical Characterizations of Pickering Emulsion Formula

Pickering emulsion formation was investigated by a polarized microscope, transmission electron microscope (TEM), and confocal laser scanning microscopy (CLSM).

Samples were observed at a 40× magnification using a polarized microscope equipped with an Olympus SC30 camera with 3.3 megapixels (Olympus BX50, Japan) at room temperature. About 10 μL of the emulsions was placed on a glass slide and covered with a coverslip; then, globules were observed. Colloidal and globule movements were also measured as follows: during each experiment, six images were obtained from 0 s to 30 s through GetIT software and were blended using Adobe Photoshop CS6 software.^28^ To determine the displacement distance of colloids, about 20 trajectories for each sample were measured with the aid of the graphic editing program ImageJ (National Institutes of Health, USA).^29^ All of the measurements were conducted in duplicate. The measurements were expressed as the average from 20 trajectories at 0 to 30 s.

For TEM, samples were first diluted to an almost clear appearance. About 15 μL of the sample was dripped onto the TEM grid and then allowed to stand for 1 min. 15 μL of Uranyless was used as a negative staining agent and then dripped onto the TEM grid. Samples were observed after 1 h of preparation using Hitachi HT7700, Japan.

The microstructure of Pickering emulsion and the colloidal network was observed using a confocal laser scanning microscope (CLSM Olympus FV1200, Japan). The sample was added with 0.1% of Nile blue staining agent and incubated for 15 min. Samples were placed on a confocal disc and covered with a coverslip. The structure was revealed with the emitted light observed at 488 for red autofluoresence of oil globules and/or 633 as gray (shown as green in the images) of basil extract as Pickering or colloidal network. In addition, other characterizations investigated in this study were particle size, conductivity, and pH using a similar method as described in Section 2.8.

### 2.10 Total Phenolic Compound (TPC) Analysis

TPC was analyzed using the Folin–Ciocalteau method using a UV–vis spectrophotometer (DU720 Beckman Coulter, USA). Gallic acid was used as the standard. The calibration curve was made by making a series of gallic acid concentrations between 60 and 110 μg/mL. Samples of each concentration were piped 50 μL and put into a test tube; then, 500 μL of Folin–Ciocalteau reagent (10%) and 400 μL of 1 M sodium carbonate 1 M were added and then incubated for 30 min. The absorbance of the solution was measured at 765 nm. TPC in the extract was determined in the same way as gallic acid. TPC was expressed as milligrams of gallic acid equivalent per gram of dry extract weight (mg GAE/g).^30^

### 2.11 Antioxidant Activity Analysis

Antioxidant activity was analyzed using the DPPH free-radical scavenging method.^30^ Ascorbic acid was used as the standard. The calibration curve was made by making a series of ascorbic acid concentrations between 1 and 4 μg/mL. Samples of each concentration were piped 500 μL and put into a test tube; then, 500 μL of 65 μg/mL DPPH solution (1:1) was added. The control was 500 μL of methanol and 500 μL of 65 μg/mL DPPH solution (1:1). The solutions obtained were incubated for 30 min in a dark room. The absorbance of the sample and control was measured at 517 nm using a UV–vis spectrophotometer.

The percentage reduction of the DPPH radical content (% inhibition, %*I*) was expressed using the following equation
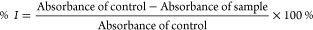
3Based on the % inhibition, IC_50_, an index for comparison of the antioxidant activities, was calculated. The value of concentration and % inhibition was plotted on the *x* and *y* axes, respectively, so that the linear regression equation is obtained
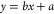
4where *y* is 50 (determination) and *x* is an antioxidant activity (IC_50_).

For the emulsions, antioxidant activity was expressed in % IC_50_. The control and standard used were the same as in the DPPH analysis. The difference was in the sample dilution, namely, at a concentration of 50 μg/mL. % IC_50_ was calculated using the following equation
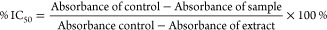
5

### 2.12 In Vitro Lipid Membrane Permeation Test

The in vitro study was carried out according to the method of previous research by Astuti et al.^31^ and Tofani et al.^32^ with modifications. The study was conducted with an artificial membrane in Franz diffusion cells. The membrane was made using Whatman paper grade 1, which was immersed with Spangler solution. Whatman paper grade 1 used is a 3 cm diameter cellulose filter with a pore diameter of 11 μm. The composition of the Spangler solution is sesame oil 20%, 15% coconut oil, 15% oleic acid, 15% white vaseline, 10% liquid paraffin, 10% palmitic acid, 5% cholesterol, 5% stearic acid, and 5% squalene. All materials were melted starting from the highest melting point, and then Whatman paper was immersed in the solution for 15 min. The paper was lifted and stored between 2 paper filters to reduce the dripping of Spangler solution.

Artificial membranes that have been prepared were weighed to determine the amount of fluid absorbed. The amount of fluid absorbed was calculated using the following equation
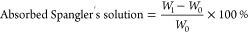
6where *W*_0_ is the weight of the membrane before treatment and *W*_1_ is the weight of the membrane after treatment. The membrane was qualified in the uniformity test if the percentage value of absorbed Spangler’s solution ranges at 102.19–131.22%.

The diffusion cell was prepared for diffusion test; about 100 mg of samples containing 2% of basil extract was mounted on the surface of the diffusion plate size; then, the artificial membrane was placed on top of it. The intake of air between the membrane and samples was avoided. 50 mL of buffer solution of pH 7.4 was prepared as a receiver solution and stirred at 60 rpm. The diffusion cell was placed in a water bath, connected to a peristaltic pump, with stirring rate and temperature maintained at 50 rpm and 37 ± 1 °C, respectively. Sampling was carried out at 30, 60, 90, 120, 150, 180, 210, and 240 min. The volume of solution withdrawn was 2 mL and immediately replaced with a new pH 7.4 buffer solution with the same volume and temperature after each withdrawal. Measurements of absorbance were made with a UV–vis spectrophotometer.

The percentage of sample diffusion (*D*) was calculated using the following equation
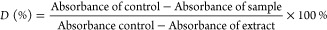
7Then, the correction factor (CF) must be calculated according to the following equation
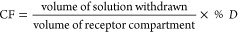
8The correction factor obtained was then calculated as cumulative values from a sampling time of 30 min until 240 min. Then, these cumulative values were added in each sampling time to the uncorrected percentage of sample diffusion. The end result is the corrected percentage of sample diffusion. A curve between the corrected percentage of sample diffusion vs time was made.

### 2.13 Cellular Uptake

Cell penetration of basil extract formulation was tested using cellular uptake analyses applied by previous research with modifications.^33^ A fibroblast 3T3 cell suspension in Dulbecco’s modified Eagle’s medium (DMEM) supplemented with 10% fetal bovine serum and 1% antibiotic/antimycotic was seeded in a confocal disc at 10^5^ cells per disc and incubated for 24 h (37 °C, 5% CO_2_). The media was discarded and replaced with the test samples, namely, the emulsion base, Pickering emulsion, and colloidal network formula mixed with Nile red and then reincubated for 1 h. The media was discharged, and the cells were rinsed with PBS three times to remove unuptake samples. Then, the cells were stained using Hoechst 33342 for nucleus staining and observed using CLSM.

### 2.14 In Vitro Antiaging Testing on Fibroblast Culture Testing

The antiaging effect of emulsion formulation was tested on UV-B-irradiated fibroblast. Collagen deposition induced by samples was tested on fibroblast with and without UV-B irradiation based on previous research with modifications.^34^ Cells (10^4^ per well) in 96-well plates were incubated for 24 h (37 °C, 5% CO_2_) and treated with UV-B exposure for 20 min. The media was discarded and replaced with the test samples (extract solutions, the emulsion base, Pickering emulsion, and colloidal network formula) at a concentration of 100, 200, 300, and 400 μg/mL in media containing 2% FBS and antibiotic/antimycotic 1% and then reincubated for 3 days. Media were discharged and rinsed with PBS three times. Collagen depositions were analyzed by adding 200 μL of 0.1% picro sirius red (Direct red 80 dye, Sigma-Aldrich) in saturated picric acid and incubated for 60 min. The cells were rinsed with 0.01 N HCl 3 times to remove the unbound dye. The collagen deposition was analyzed quantitatively using light microscope imaging and qualitatively by dissolving the deposited dye in 150 μL of 0.5 N NaOH at room temperature for 30 min with gentle shaking, and the optical density was measured at 540 nm using a plate reader. The relative collagen expression to the base formula was shown.

### 2.15 Statistical Analysis

All experiments were performed in triplicate, and the results were expressed as mean ± standard deviation (SD). Data were analyzed by analysis of variance (ANOVA) using Minitab 20.2 software (Minitab LLC, USA). If the results of ANOVA showed *p* values lower than 0.05, or significantly different, then proceed using the Tukey multiple comparison test.^35^

## 3 Results and Discussion

### 3.1 Characterization of Basil Extract (BE)

In this study, the active components of basil leaves (*O. americanum, L.*) belonging to Lamiaceae family were extracted using an ultrasonic bath in a solvent mixture of water, 70% ethanol, and citric acid. The extraction method was modified from Bezerra et al.,^36^ which yielded an amphiphilic extract with high surface activity produced from a hydroalcoholic solvent in the range of 50–80% using ultrasonic agitation. However, previous research revealed that the most abundant *O. americanum* leaf extract components were phenolic compounds present in aqueous extracts;^37^ therefore, we added citric acid as a hydrogen bond donor^38^ to increase the extraction of basil leaves using 70% ethanol. Furthermore, citric acid was also proposed as a solid diluent for freeze-dried basil extracts. The use of ultrasonic agitation, which is capable of producing acoustic cavitation, can also increase the extraction yield of phenolic compounds.^39^

The dried extract of basil leaves was analyzed qualitatively and quantitatively for its phytochemical profile (Table 2). The yield of basil extract powder calculated from wet leaves was 1.52 ± 0.03%, where the powder in admixture with citric acid had an extract content of 53.92 ± 0.78%. Quantitative analysis showed that the basil extract contained total phenolic compounds with strong antioxidant activity as measured by the radical scavenging effect on DPPH with an IC_50_ value of 47.04 ± 2.96 μg/mL. These data were not significantly different from the basil extract powder extracted using 70% ethanol without citric acid (*p* > 0.05). This result indicates that basil extract has strong antioxidant activity compared to the activity of some Lamiaceae species, which were shown to have IC_50_ > 50 μg/mL.^40^ The strong DPPH radical scavenging of American basil water extract was found by Zengin et al.^37^ Basil extract containing citric acid was used for further research, considering the ease of dissolving the extract in water at high concentrations.

**Table 2:** Qualitative and Quantitative Analyses of Basil Extract (BE)^a^

qualitative		quantitative	
phenolic compound	+	a. DPPH scavenging/IC_50_ (μg/mL)	
tanin	+	- Et-CA	: 47.04 ± 2.96^b^
flavonoid	+	- Et70%	: 44.20 ± 1.38^b^
saponin	+	b. total phenolic content (mg GAE/g)	
triterpenoid and steroid	–	- Et-CA	: 89.4 ± 0.29^c^
		- Et70%	: 95.2 ± 1.42^c^
		c. total flavonoid content (mg QE/g)	
		- Et-CA	: 7.0 ± 0.20^d^
		- Et70%	: 3.8 ± 0.10^d^

a Note: Et-CA: a mixture of 70% ethanol and 30% citric acid. 1%; Et70%: ethanol 70%.

Meanwhile, total phenolic compounds were 10–15 times higher than the total flavonoid content. This is supported by previous reports,^37,41^ which stated that the high radical scavenging activity of water leaf extract resulted from the high content of total phenolic acid. The qualitative test for the phytochemical profile showed additional components in the form of moderate tannin and saponin, in addition to the previously mentioned components, which were also in accordance with the previous study.^37^ The presence of saponin can potentially support the solid particle stabilization of phenolic compounds in Pickering emulsion by filling the gap between particles on interfacial oil globules.^42^

### 3.2 Characterization of Phenolic Compounds

**Figure 1:**
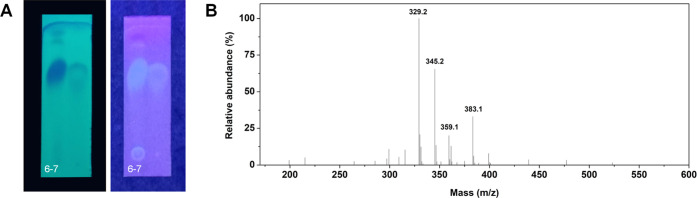
Thin-layer chromatography (TLC) (A) and LC-MS/MS analysis of the phenolic compound of *O. americanum* L. (B).

The analysis of phenolic compounds from basil extract began with TLC, which indicated fractions 6–7 as fractions to be further analyzed (fractions containing phenolic compounds) (Figure 1A). Furthermore, analysis using LC-Q-TOF-MS/MS produced the four highest peaks with *m*/*z* 329.2, 345.2, 383.1, and 359.1 (Figure 1B). Peak identification was performed by literature search and showed characteristics as shown in Table 3. The phenolic compounds have varying characteristics related to their hydrophobic nature represented as Log *P* values and potential hydrogen bond groups.

**Table 3:**
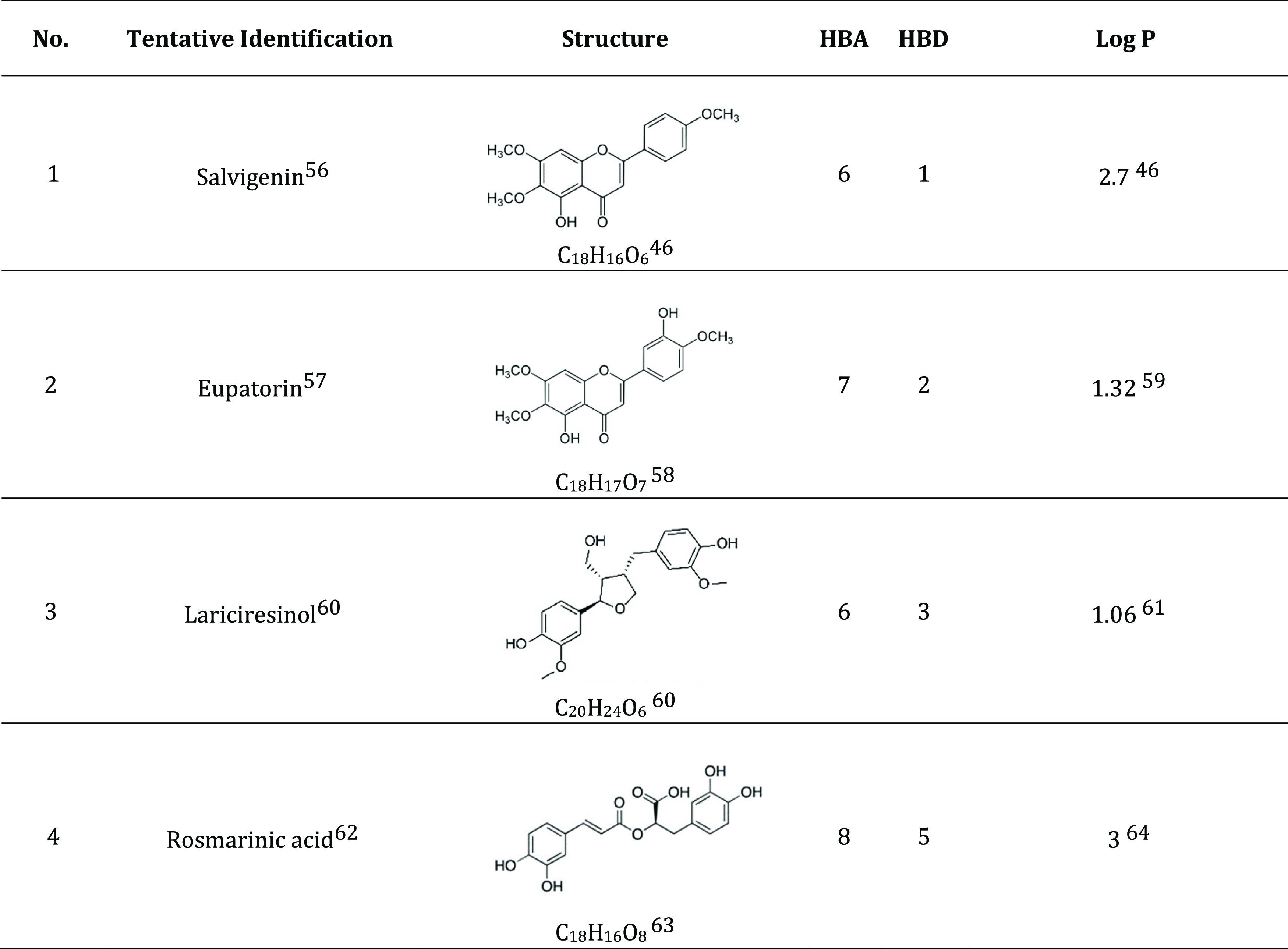
Tentative Identification of Major Phenolic Compound of Basil Extract LC-MS/MS Result^56−64^

Log *P* is a quantitative representation of hydrophobicity as measured by the ratio of the concentration of unionized compound at equilibrium between the hydrophobic and aqueous phases, which usually consists of *n*-octanol/water.^43,44^ A compound is categorized as hydrophobic if the Log *P* value > 1.^45^ Rosmarinic acid, salvigenin, eupatorin, and lariciresinol with Log *P* values varying from 1 to 3 are listed in Table 3. The phenolic compounds of basil extract have a Log *P* value > 1, which indicates that they are hydrophobic.^45^ Salvigenin is relatively insoluble in water based on its Log *P* value of 2.7^46^ and has less HBA and HBD than rosmarinic acid. However, salvigenin has the potential as a solid particle stabilizer as shown by Luo et al.^21^ who demonstrated the emulsifying effect of tiliroside with Log *P* 2.7 in stabilizing O/W Pickering emulsions. Furthermore, eupatorin (Log *P* value 1.32) and lariciresinol (Log *P* value 1.06) are relatively insoluble in water^47,48^ and can interact with water through hydrogen-bonding sites;^48^ hence, they can stabilize the emulsion by their presence at the oil–water interface.

Rosmarinic acid has a high calculated Log *P* value due to numerous hydroxyl groups that can interact with octanol.^49^ However, the high number of hydrogen-bonding donor sites, namely, hydroxyl and carboxyl groups of rosmarinic acid, does not indicate that rosmarinic acid is highly soluble in oil.^49^ The hydrophobic parts (ring groups) tend to interact with the oil phase, while the hydrophilic parts tend to interact with the water phase.^49^ The increasing number of hydrogen-bonding donor groups causes the hydrophobicity decrease in the order salvigenin > eupatorin > lariciresinol > rosmarinic acid. Therefore, basil extract with varying hydrophobicity of phenolic compounds is a promising emulsion stabilizer. The relative Log *P* value of phenolic compounds can be one of the factors that support their functionality to form a more stable emulsion.^21^

### 3.3 Characterization of Surface Activity, Conductivity, and Potential Colloidal Formation

Emollient formulations require an emulsifier to provide long-term stability, whereas surfactants are currently widely used because of their surface activity effects. However, an increased emphasis on going back to nature is proposed with increasing evidence of surfactant side effects such as irritation and toxicity.^15,16^

In order to address the current needs, various studies in the last decades have introduced solid particle-stable emulsions, called Pickering emulsions. Due to the amphiphilic nature of phenolic compounds, it has been proposed as a promising target of Pickering emulsion stabilizers, in which the phenolic ring groups will be adsorbed on the interfacial oil droplets, while the polyhydroxyl groups form hydrogen bonds with the aqueous phase.^50^ Therefore, the presence of hydrophobic and hydrophilic parts makes the phenolic compound act as a surface-active agent.^49^ While phenolic compounds have been prepared in Pickering emulsions, most studies were carried out by adding other solid particles as costabilizers, such as starch,^51^ zein protein,^52,53^ or whey protein,^54^ and only a few studies employed phenolic solid particles as the main stabilizer.^55^

We proposed a Pickering emulsion green formulation of the basil extract using the emollient formulation. SFO and urea were selected as emollients and moisturizers, respectively, with two types of hydrophilic and hydrophobic humectants, namely, glycerol and hexylene glycol. The last humectant was involved in this study to optimize the solidification of phenolic compounds in stabilizing the interfacial oil droplets. This is since hexylene glycol is miscible with many solvents that are used in cosmetics and has been applied to optimize Pickering emulsion stabilization.^65^ There is a concern for fine-tuning the mixing steps and preparation technology by considering the use of hydrogen bond donors in the formulation using glycerol and urea.^38^ These substances have the potential to bind phenolic compounds to dissolve in aqueous external phases, which can negatively impact PE stabilization at inappropriate amounts. We also studied the possibility of adding a surfactant commonly present in cosmetics (Tween 20) to see a positive effect in emulsion stabilization by modifying the polarity of the oil^66^ or conversely destabilizing Pickering emulsion by delaminating solid Pickering particles from the interfacial globules.^67^

Solid particle deposition of the basil extract components on the interfacial globules became the rate-limiting step in constructing a stable Pickering emulsion. We proposed a facile Pickering emulsification by controlling in situ colloidal formation. The basil extract was produced using a solvent mixture of 70% ethanol–30% citric acid, containing high amounts of amphiphilic polyphenols bound by hydrogen bonds with citric acid.^38^ Although the phenolic compound from basil extract has limited solubility in water, adding citric acid increased its solubility at high concentrations (200 mg of basil extract in 2 mL of water) at room temperature. This saturated solution facilitated the deposition of colloidal particles (in situ solidification) upon contact with nonpolar oil globules through the interaction of the ring groups.

Although necessary to increase the solubility of phenolic compounds in water, excess hydrogen bonding can cause the diffusion of solid particles into the aqueous phase, thereby reducing their ability to stabilize solid particles. Reducing the surface dielectric constant around the aqueous extract was attempted to increase the hydrophobicity, thereby favoring the aggregation of colloidal particles in the water phase^68^ leading to in situ particle aggregation required to form oil-in-water Pickering. For this purpose, hexylene glycol was added to the saturated solution of the basil extract to induce the formation of colloidal particles. This glycol, termed 2-methyl-2,4-pentanediol (MPD), is commonly used to modify the aqueous dielectric constant to precipitate biological macromolecules.^69^ This substance has also been successfully applied to form lysozyme Pickering emulsion in the presence of an anionic surfactant used for solidification.^65^ Particle-modified hydrophilicity using glycerol has also been applied to improve the stability of Pickering emulsions.^70^

We analyzed the hydrophilicity and hydrophobicity of formulation components by measuring surface tension and interfacial tension using the Du-Nuoy ring tensiometer. The results are shown in Table 4.

**Table 4:** Surface Properties of Formulation Components

A. Surface Tension (mN/m)	
basil extract (BE)	35.2 ± 0.05
sunflower seed oil (SFO)	34.4 ± 0.11
Tween 20 (T)	36.6 ± 0.05
hexylene glycol (H)	29.9 ± 0.05
glycerol (G)	63.3 ± 0.23
BE and hexylene glycol mixture (BE-H)	35.5 ± 0.09
BE and glycerol mixture (BE-G)	36.2 ± 0.11

B. Interfacial Tension (mN/m)	
SFO with hexylene glycol (SFO/H)	2.0 ± 0.66
SFO with glycerol (SFO/G)	15.3 ± 0.06
SFO and Tween 20 mixtures with BE (SFO-T/BE)	6.2 ± 0.17

Basil extract, SFO, and Tween 20 had the same hydrophobicity properties with a surface tension of around 34–36 mN/m. This is predicted from the variation in hydrophobicity of the mixture of the four major amphiphilic compounds in the basil extract to produce a mixture that is surface-active equivalent to Tween 20. With the solubility properties mentioned above, they act as solid particle stabilizers at the oil–water interface in O/W Pickering emulsion. In general, phenolic compounds are known to be present at the oil–water interface in both W/O and O/W emulsions,^21,71^ but since this emulsion is intended for topical antiaging cosmetics (not greasy), an O/W emulsion was chosen.

Meanwhile, hexylene glycol is slightly hydrophobic and glycerol is more hydrophilic according to their surface tension values. Surprisingly, the mixture of basil extract and hexylene glycol had the same surface tension value as the mixture of extracts with glycerol, and these values were also similar to the previous three liquids. In terms of interfacial tension, which can be used to predict liquid miscibility, hexylene glycol has the lowest value, implying that it has better miscibility with SFO than glycerol and basil extracts.

Although all humectants and basil extracts were separated with SFO at room temperature, they could be heated to increase their miscibility during emulsification. To obtain maximum contact of the basil extract components with SFO, half the amount of hexylene glycol was mixed with the extract solution and the other half was mixed with SFO during heating. It is also possible to mix glycerol with SFO because its interfacial tension is lower than the surface tension of the oil. These data indicate that all formulation components, except urea, have the same hydrophobicity and can be mixed during emulsification. Preliminary data (unpublished) showed that urea could not be mixed during the emulsification process because it caused demulsification. Hence urea was added to the emulsion after the emulsification process; it will be discussed later in the Section 3.7 on particle motion.

**Figure 2:**
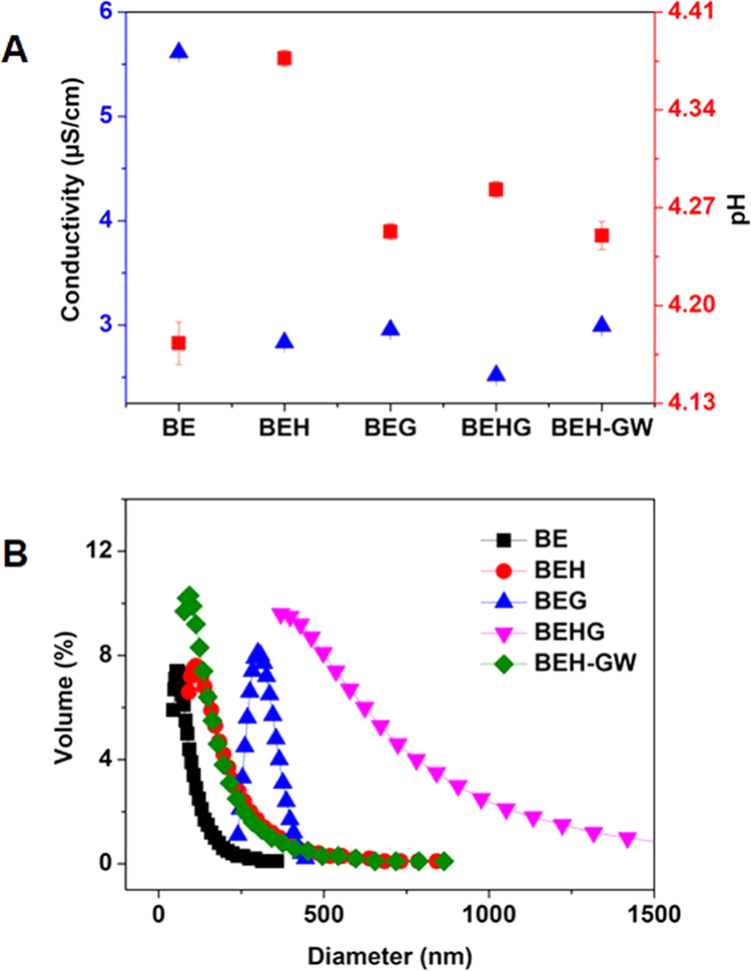
Conductivity data (A) and colloidal particles (B) of basil extract mixed with hexylene glycol and/or glycerol in water.

In addition to lowering the surface and interfacial tension by mixing the oil and basil extract solutions with humectants, we also analyzed their effect on conductivity (Figure 2A). The basil extract solution (2% w/v) in water had a conductivity of about 5.613 ± 0.007 μS/cm, which was much higher than the conductivity of 1% citric acid (2.16 ± 0.03). This implies that the phenolic compound as the main component of basil extract is amphiphilic, which is indicated by a relatively high conductivity value and low surface tension (similar to SFO). We confirmed this prediction by measuring the conductivity of the basil extract with the addition of 1% citric acid, which showed only a slight increase in its value to 5.889 ± 0.0007.

Surprisingly, the addition of hexylene glycol and/or glycerol to the basil extract solution decreased the conductivity significantly to about 2 μS/cm. The change in conductivity was also accompanied by an increase in the pH of the extract. We hypothesized that preconditioning basil extract with humectant prior to emulsification becomes a great alternative to minimize diffusion to the external aqueous phase and conversely increase the hydrophobicity leading to prolongation of colloid presentation to the interfacial oil phase and subsequent adsorption for Pickering emulsion formation.

As shown in Figure 2B, the colloidal particles formed in a solution of basil extract in water have a very small size of 1.7 nm; PI 0.295 (the distribution curve not included) and slightly increased the size to 82 nm; PI 0.22 (BE) with heating 70–80 °C. As expected, the addition of hexylene glycol or glycerol led to the formation of colloids with an increase in size to 173 nm; PI 0.24 (BEH) and 313 nm; PI 0.06 (BEG), respectively. It is suggested that the addition of humectants provides suitable conditions for the formation of colloidal particles of basil extract. However, mixing with hexylene glycol and glycerol together could not be carried out, which resulted in a very large particle size (BEHG 676 nm: PI 0.40). Meanwhile, the addition of glycerol to the aqueous phase of the mixed hexylene glycol–basil extract caused a slight decrease in particle size (BEH-GW 147 nm; PI 0.40), which may be due to the colloid redissolving by the hydrogen-bonding effect of glycerol to solubilize phenolic compounds in water.^38^ These conditions were further optimized in the Analysis of the Emulsification Process section.

### 3.4 Analysis of the Emulsification Process

**Figure 3:**
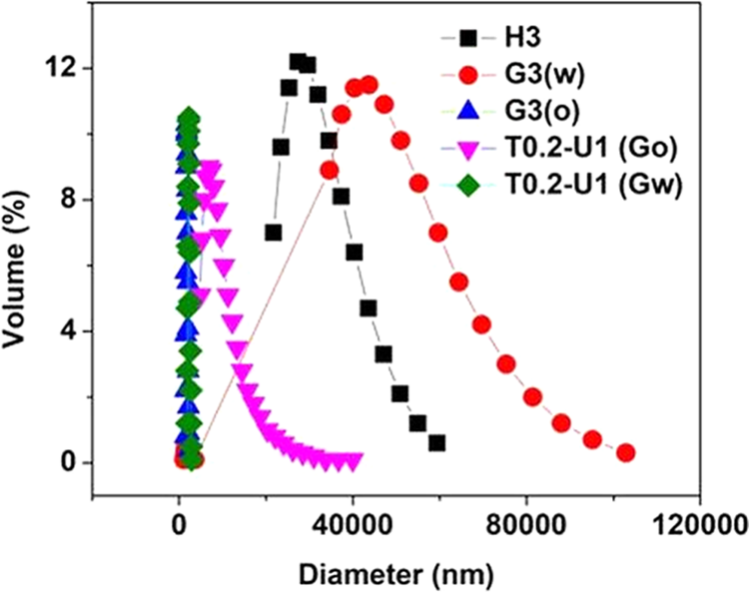
Initial prediction of the globule size of emulsion with variations of humectants mixed in the oil phase and/or water phase.

Pickering emulsion was made using 2% of basil extract by adding a single or mixed humectant, together with/without the costabilizer Tween 20 and urea. Emulsion globules were measured using a particle size analyzer as shown in Figure 3. The addition of a single humectant, either 3% hexylene glycol (H3) mixed with basil extract or 3% glycerol in aqueous phase (G3 w), without the addition of Tween 20, could not completely emulsify SFO (3%) with very large grains (>5–100 μm). A thin layer of floating coalescence oil was found. However, the use of single glycerol mixed in oil phase (G3o) improved emulsification with globules in the range of 1400–2300 nm, but some globule coalescence was still found. It is suggested that glycerol can modify the hydrophilicity of the oil and provide hydrogen bonding for the adhesion of phenolic compounds,^38^ thereby enhancing the adhesion of solid particles.

Then, we prepared the emulsion by supplementing 0.2% Tween 20 with hexylene glycol added in the basil extract and SFO, along with the addition of glycerol in the oil. Urea (1%) was added after emulsification to further stabilize the globules by forming hydrogen bonds with phenolic compounds, as demonstrated in the extraction method of deep eutectic mixtures reviewed by Rente et al.^38^ This formula can emulsify oil better but with a slight increase in globules in the range of 4–20 μm (T0.2 U1 Go). This may be the result of the oil being too hydrophilic, which limits the adhesion of solid particles.

Finally, we emulsified the extract using the latter method, but with glycerol added in the external aqueous phase (T0.2 U1 Gw). This was the best preparation method, which can completely emulsify SFO with the smallest granules, suggesting the use of preconditioned basil extract in a hydrophobic environment and a balanced hydrogen bonding in both the oil and water phases. To conclude, Pickering emulsion was prepared by heating a mixture of aqueous dispersion basil extract with hexylene glycol and sunflower oil mixtures with hexylene glycol with/and without the addition of Tween 20. Then, it was mixed and homogenized with the aqueous phase containing glycerol at 9000 rpm for 3 min. Finally, Pickering emulsion was stabilized using various concentrations of urea with homogenization for 1 min.

Colloidal particles were formed by in situ solidification of a saturated solution of basil extract (10% w/w) in contact with the oil phase.^72^ The ring groups of phenolic compounds tend to bind to the hydrophobic surface of the oil droplets, while the hydroxyl groups form hydrogen bonds with glycerol and water molecules surrounding the oil droplets.^70^ Therefore, the colloidal particles provide a strong reduction of the interfacial oil surface and stabilize the emulsion. This condition can be correlated with small molecule surfactants with their amphiphilic structure in stabilizing the interfacial surface. Interestingly, the strong bonding of the colloidal particles with the oil resulted in smaller granules and a more stable emulsion.^73^

### 3.5 Pickering Emulsion Construction of Basil Extract

**Figure 4:**
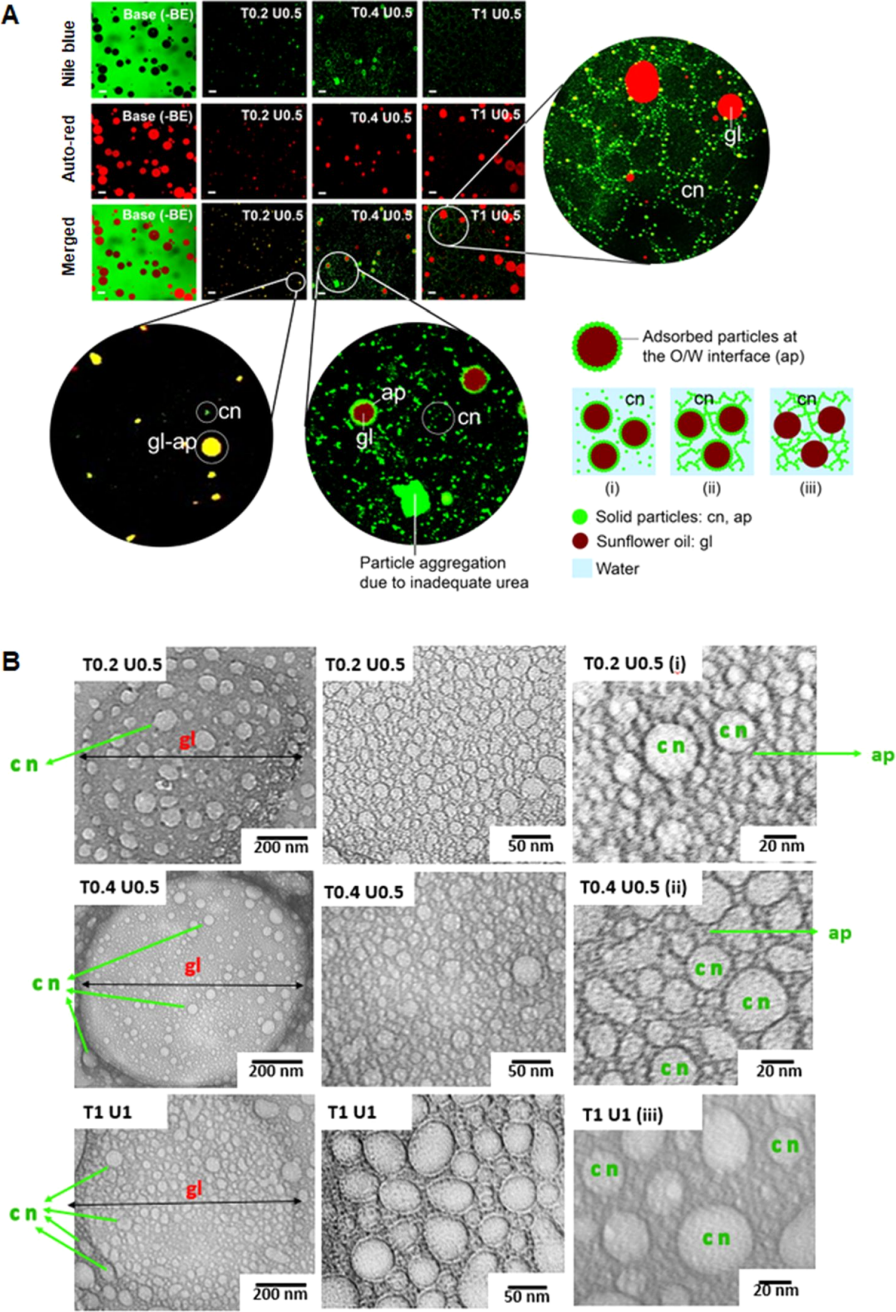
Microstructure of the O/W Pickering emulsion and colloidal network found in basil extract observed by the approved confocal microscope using Nile blue fluorescence staining of phenolic compounds and auto-red oil globules. Scale bar is 1 μm (A) and transmission emission microscope (B). System behavior: (i) Pickering emulsion and colloidal network systems (mixed system) with inadequate urea; (ii) mixed system with adequate urea; and (iii) colloidal network system. The approximated colloidal particle size was determined by imageJ in the range of (i) 5.7–22.41 nm, (ii) 4.0–29.7 nm, and (iii) 8.30–31.54 nm. Note: gl, globule; cn, colloidal network; and ap, adsorbed particle.

Basil extract Pickering emulsions were characterized by confocal and transmission electron microscopies as depicted in Figure 4A,B. The microstructure of the Pickering emulsion (Figure 4A) was obtained by observing the overlap of the green color of the Nile blue-labeled phenolic compounds with the red autofluorescence of the oil droplets to form yellow globules. The emulsion base without basil extract (Bs T1 U1) did not produce a yellow color globule, where a green color covered the external water phase homogeneously and the globules remained red. In the T0 U1 formula, there is a perfect overlap of yellow color on the surface of the globules; this represents colloidal particles adhered on the oil surface, suggesting the Pickering emulsion structure. Surprisingly, in the Tween 20-added formula (T1 U1), it was observed that the globules remained red, but the colloidal particle network was found in the external aqueous phase.

This is an interesting finding that the difference in the concentration of Tween 20 supplementation on o/w sunflower oil emulsion stabilized with phenolic compounds, along with the addition of glycerol, hexylene glycol, and urea, displayed two emulsion systems, namely, Pickering emulsion (T0.2 U0.5) and colloidal particle network (T1 U1). In addition to variations in the Log *P* value of phenolic compounds contained in colloidal particles, the concentration of Tween 20 also affects the system’s behavior. The low concentration of Tween 20 maximized the adsorption of colloidal particles from amphiphilic compounds of basil extract at the oil–water interface, especially in the presence of salvigenin and rosmarinic acid components with high Log *P*. Also, a few colloidal particles were found that formed structures in the aqueous phase (Figure 4A). The particle stabilizer’s adsorption system at the oil–water interface is called the Pickering emulsion dominance system.

In conditions of excess Tween 20 concentration, it can inhibit the adsorption of colloidal particles into the oil–water–oil interface and dominate the interface. The presence of amphiphilic compounds with a Log *P* of around 1 will form colloidal particles dispersed in the aqueous phase. In this study, we prevented this aggregation by adding urea as a hydrogen bond donor that can bind to hydrogen bond acceptors in the colloidal particles of basil extract, namely, eupatorin and lariciresinol. Colloidal particles not adsorbed at the oil–water interface will form a network structure in the water phase (colloidal network-dominated system). At the intermediate concentration of Tween 20, a mixed system was found (Figure 4A). These results align with research conducted by Pichot et al.^74^ and Zhao et al.^53^

A more detailed morphology can be seen from the TEM image (Figure 4B), which shows the Pickering emulsion and colloid network systems, as well as both. The existence of the two systems cannot be significantly distinguished and can only be categorized based on their dominance. In the Pickering emulsion system, colloidal particles will still be found in the aqueous phase, such as in TEM T0.2 U0.5 with a scale of 200 nm. At low magnification (200 nm scale bar), colloidal particles can be seen in the T0.2 U0.5 Pickering emulsion system. However, the dominant system can be more detailed at higher magnification (50 nm scale bar). On the 50 nm scale, it can be seen that the surface coverage of the adsorbed particles (denoted by ap = adsorbed particles) is very high, with variations in the shape and size of the particles in a wide range (still below 50 nm) so that they can completely cover the surface oil to prevent globule coalescence. Meanwhile, the unadsorbed particles were in the aqueous phase as insoluble particles (denoted by cn, colloidal network). However, the particles tend to aggregate^21^ as seen in formulas T0.4 U0.5, so that sufficient urea is needed to stabilize the emulsion and form a network structure through hydrogen bonds between urea and particles. Confocal images revealed colloidal networks in the aqueous phase more clearly compared to TEM imaging.^75^

The aggregations of colloidal particles were found in the water phase at a urea concentration of 0.5% (83 mM). Colloidal particle aggregation containing these phenolic compounds in water may also be associated with low solubility.^21,76^ Therefore, in this study, optimization of the concentration of urea can provide hydrogen bond donors in colloidal particles with the carboxyl group of rosmarinic acid and a more significant number of hydrogen bond acceptors (HBAs) (salvigenin (HBA 6), eupatorin (HBA 7), lariciresinol (HBA 6), and rosmarinic acid (HBA 8)), thus increasing the interaction of particles with the aqueous phase and preventing particle aggregation in the aqueous phase to form a colloidal network. In the next section, optimization of urea concentration will be carried out.

### 3.6 Influence of Tween 20 Addition on Pickering Emulsion

**Figure 5:**
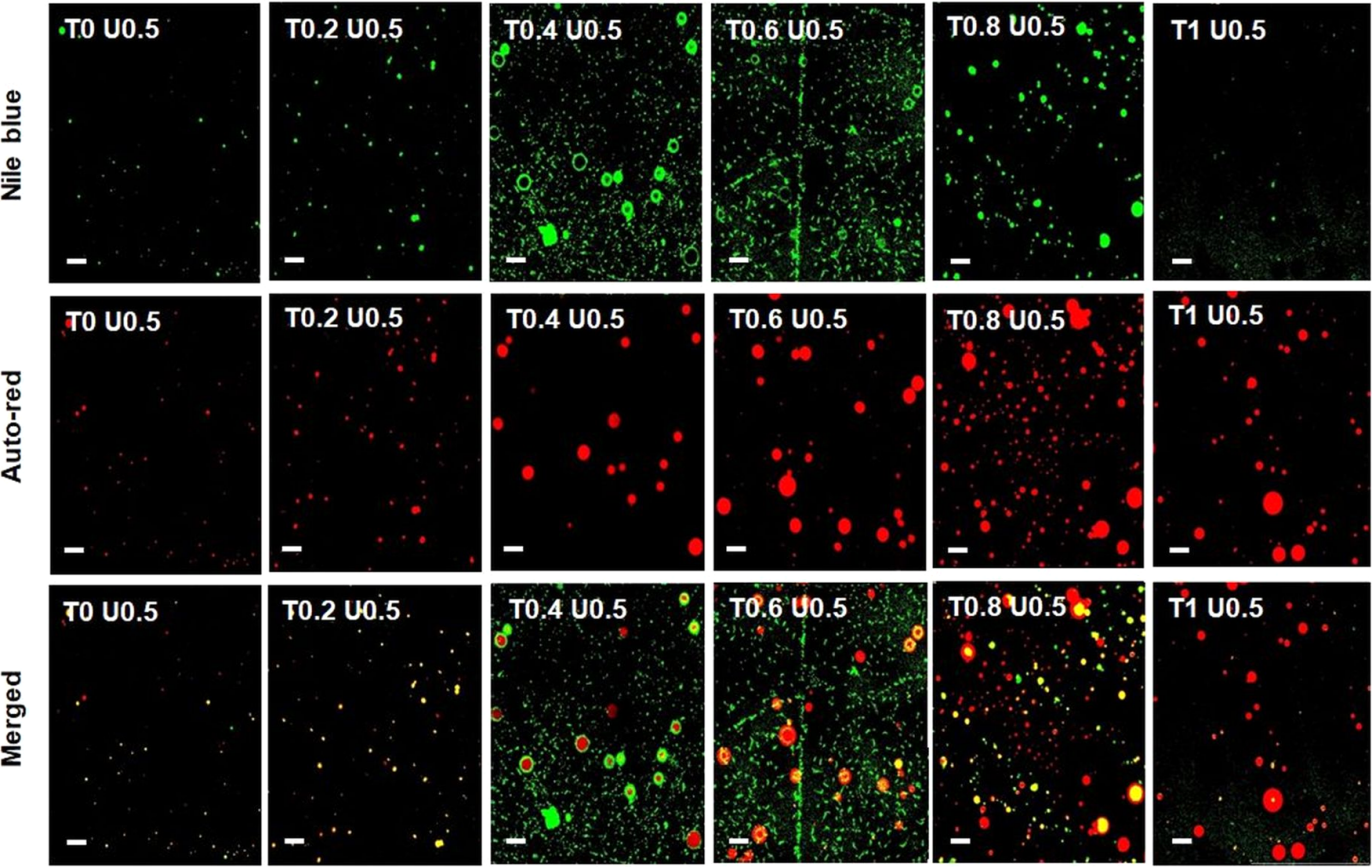
Confocal microscope observation of the O/W Pickering emulsion microstructure at various Tween 20 supplementations. Green fluorescence shows Nile blue emission, red fluorescence indicates autofluorescence emission resulting from sunflower oil globules, and yellow fluorescence shows an overlapping green and red fluorescence in the same location. Scale bar is 1 μm.

As previously mentioned, the formula without Tween 20 (T0 U1) had some red globules found in the confocal images (Figure 4A), indicating incomplete stabilization of solid particles. This was also supported by visual observations, where some coalescence globules were found on the surface of the emulsion. This could be due to insufficient hydrophilicity of the oil phase, which hindered contact with the extract. We added Tween 20 as a costabilizer, as has been applied by other researchers.^53,66,77^ The microstructure of the Pickering emulsion at various concentrations of Tween 20 is shown in Figure 5.

Pickering emulsion was indicated by the presence of yellow globules in the formula^42^ with Tween 20 of 0 (T0 U0.5) and 0.2% (T0.2 U0.5) added. The addition of a higher amount of 0.4–0.8% resulted in more color and size variation of yellow and red globules, as well as green colloidal particles. This indicates that there is a dynamic change in the formation of solid particles in situ upon contact with the oil phase and their adsorption, as well as the competition with Tween 20 on the surface of the globules. The more Tween 20 bonded to the surface of the globules, the more polar heads would prevent solid particles from adhering to the oil globules.^67^ It is therefore found that many red globules were surrounded by incomplete green solid particles, along with a mixture of yellow and red globules. The decrease in the number of large green particles was seen in Tween 0.8% (T0.8 U0.5).

Interestingly, the green colloidal particle size with a homogeneous distribution at a very small size was found in the formula T1 U0.5. It can be predicted that the complete adsorption of Tween 20 on the globule surface prevents the formation of colloidal particles in situ, so that there is no change in the size of the colloid formed during heating of the extract with hexylene glycol. The morphology of the emulsion has changed from a Pickering emulsion to a network of colloidal particles with different movements and behaviors.^75^

### 3.7 Influence of Tween 20 and Urea Addition on the Characteristics of Pickering Emulsion and Colloidal Particle Network

It is generally known that Pickering emulsions are stabilized by hydrophobic interactions to attach solid particles to the interfacial oil phase and hydrogen bonds to bind to water.^78,79^ The extent of hydrogen bonding enables network formation that limits globule movement and collision for strengthening their integrity and preventing coalescence.^80,81^ Urea is a potent moisturizer^7^ that can be added to antiaging emollient formulations, so we observed here the effect of urea on the Pickering emulsion characteristic. In this regard, urea may affect the formation of hydrogen bonding between Pickering particles and the external aqueous phase due to its nature as a hydrogen-bonding donor that can bind phenolic compounds strongly, as is the case with the use of citric acid in the deep eutectic solvent extraction method.^38^

The carboxyl groups in rosmarinic acid and the hydroxyl groups in salvigenin, eupatorin, lariciresinol, and rosmarinic acid can form hydrogen bonds, especially with urea.^82,83^ In addition, the carboxyl group can form two parallel hydrogen bonds,^84^ thus allowing a reasonably strong bond with urea that have also multiple HBD and HBA sites, namely, the carbonyl and the primary amine groups.^83^ Urea was incorporated into the formulation after the emulsification process to provide the required hydrogen bonds to balance the hydrophobic bonds generated by the solid Pickering particles adsorbed on the interfacial oil droplets. It can be explained that the addition of urea to the formulation prior to the emulsification process resulted in demulsification, as opposed to the adsorption of solid Pickering particles due to the binding effect of phenolic compounds by urea to remain in the water.

Apart from variations of Tween 20 added, we observed the effect of urea addition at various concentrations on the characteristics of two different emulsion systems, namely, (i) Pickering emulsion system at 0.2% Tween 20 addition with solid particle stabilization and (ii) colloidal particle network system at 1% Tween 20 as shown in Table 5. Among the characteristics observed were particle size, electric conductivity in correlation with particle movement, and the evidence of colloidal network formation. The pH values of all of the formulations were in the range of 4.2–4.4.

**Table 5:** Average Globule and Colloidal Size of 0.2% Tween 20 and 1% Tween 20 Formulations at Various Concentrations of Urea Correlated with the Size Distribution from Day 1 to 30^a^

	globule and colloidal size					globule and colloidal size					
	day 1		day 30			day 1			day 30		
sample	*D* (nm)	PI	*D* (nm)	PI	sample	*D* colloid (nm)	*D* globule (nm)	PI	*D* colloid (nm)	*D* globule (nm)	PI
T0.2 U0	163.3	0.295	644.6	0.294	T1 U0	138.6	256.3	0.366	267	478.8	0.302
T0.2 U0.5	113.1	0.266	467.5	0.465	T1 U0.5	179.1	328.1	0.461	180.1	316.9	0.318
T0.2 U1	141.5	0.468	550	0.447	T1 U1	110	203.8	0.371	73.8	477	0.447
T0.2 U1.5	220.4	0.293	752.6	0.252	T1 U1.5	116.2	221	0.325	129.4	241.3	0.327
T0.2 U2	3251.8	–0.232	1234.6	0.261	T1 U2	82.7	519.4	0.276	294.9	563.6	0.465

a Note: D, diameter; PI, polydispersity index.

The particle size of the emulsions measured using a light scattering particle size analyzer is presented in Table 5. In the Pickering emulsion system (T0.2), the addition of 0.5% urea (T0.2 U0.5) resulted in the smallest particle size, which was 113.1 nm; PI 0.266. It is within the designed particle size range <200 nm to obtain optimal skin permeation to viable epidermis area.^85^ The addition of urea up to 1.5% to the Pickering emulsion in which the solid particles were attached with strong hydrophobic bonds to the interfacial oil globules did not result in changes in the structure between the globules. On the other hand, T0.2 U2 with the addition of 2% urea produced a very large particle size (3251.8 nm, PI −0.232). This implies that a large globule network has been formed by hydrogen bonding of urea, and further investigation is needed on its effect on emulsion stability.

The effect of adding urea to the formula containing 1% Tween 20 showed an opposite trend of changes in particle size, where the size of T1 U0.5 (*d*50% = 328 nm *D* colloids = 179.1 nm and *D* globules = 328.1 nm, PI 0.461) was larger than T1 U0, T1 U1, and T1 U1.5. Different characteristics are found in T1 U2, where the colloidal particle network is small (82.7 nm), which were indicated by the successful addition of urea. However, the oil globules were 519.4 nm, PI 0.276, related to the urea being too high. It removed colloidal particles that were adsorbed at the oil–water interface (Pickering emulsion system) so that they were not strong enough to stabilize the emulsion. Nonetheless, the increase in urea up to 2% caused colloidal particles to be attracted to the water phase, leading to the escape from the interface in the Pickering system and the colloid network systems. The release of colloidal particles from the oil–water interface made the globules easily aggregate, thereby increasing the droplet size on the 30^th^ day (Table 5). It is not a good choice for adding high concentrations of urea.

Pickering emulsions usually use an electrolyte, which plays a similar role to urea in this study. In specific concentrations, it will stabilize the Pickering emulsion, but excessive additions will cause the opposite effect. Interparticle interactions can be regulated by adding variations in salt concentration in the water phase, from repulsive to attractive, as the salt concentration increases.^86^ High concentrations of NaCl can reduce the Debye length or the electrostatic repulsion between particles so that the particles will aggregate.^87,88^

**Figure 6:**
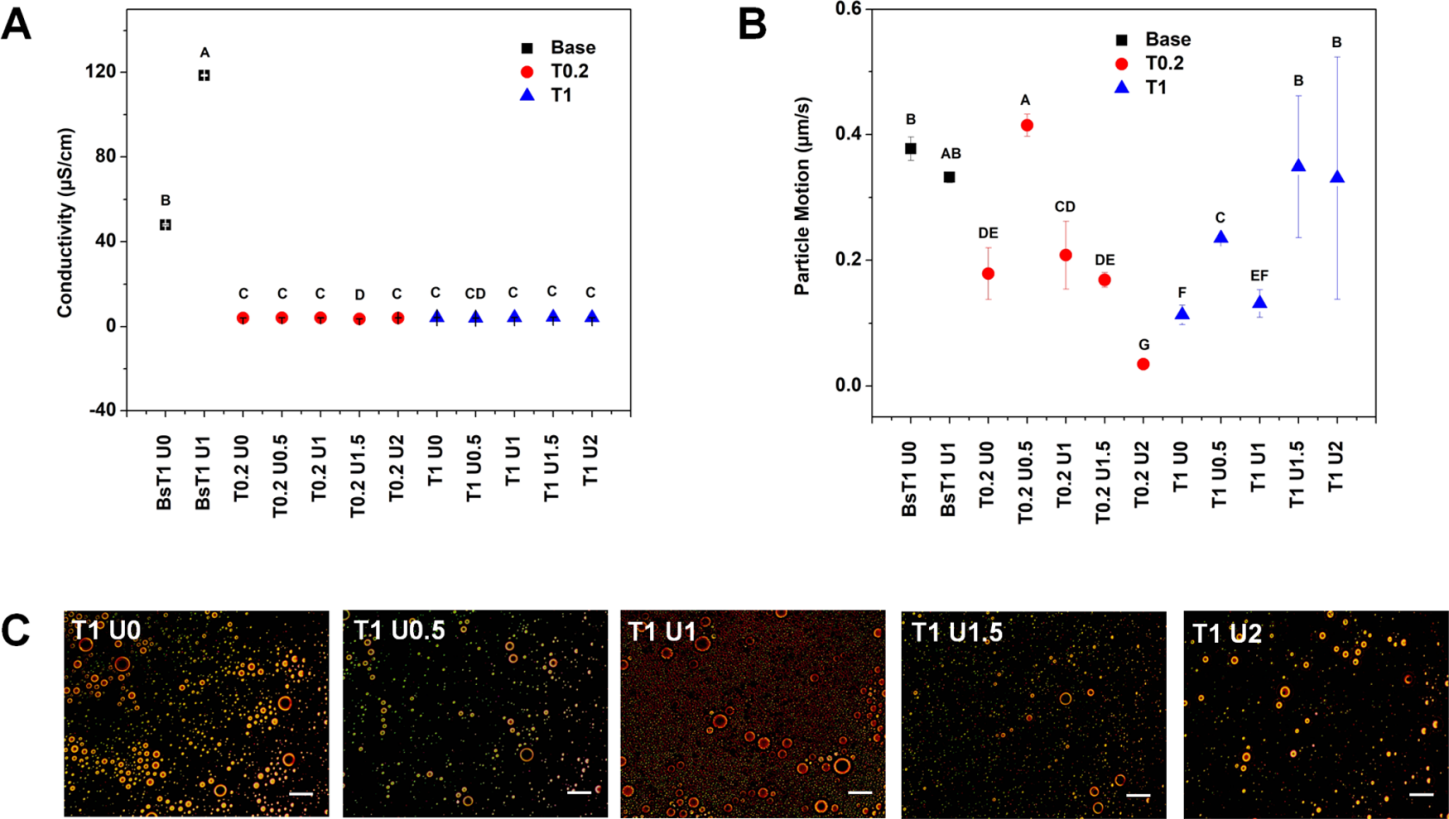
Electric conductivity of the formulation (A), particle movement (B), and colloidal network pattern of 1% Tween 20 were observed using a polarized microscope. Scale bar is 1 μm (C).

The addition of urea to the T0.2 and T1 formulas did not significantly change the electric conductivity, which was at the range of 4.069–4.138 μS/cm (Figure 6A). However, the trend of changes in particle size correlated with changes in particle motion (Figure 6B). Among the T0.2 formulas, the addition of 0.5% urea to the T0.2 U0.5 formula showed the highest particle movement (0.414 μm/s, *p* < 0.05) compared to the T0.2 U0 formula without urea (0.179 μm/s).

In contrast, almost no particle motion was observed in the formula T0.2 U2 (0.03 μm/s). These data demonstrate the benefits of adding urea to provide hydrogen bonding, which facilitates particle movement avoiding globule aggregation. It correlates with the smallest particle size of the formula T0.2 U0.5, whereas the addition of very high urea resulted in very high hydrogen bonds so that the globules remained fixed, thereby inhibiting globule movement and resulting in a very large size of the formula T0.2 U2 (3251.8 nm, Table 5).

The behavior of the globules in the T1 formula is very different. Slow globule movement in the formula without urea addition of the T1 U0 formula (Figure 6B) resulted in globule aggregation (Figure 6C). Increasing urea to 0.5% increased globule movement but decreased again with the addition of 1% urea. This can be seen in Figure 6C, which shows fixed globules within the colloidal particle network environment, and these globules are surrounded by fine particles that are constantly moving around the globules. This phenomenon was also observed by French et al.^87^ who observed the exchange of stabilizer particles between globules. Interestingly, there was a very high increase in droplet movement on the T1 U1.5 and T1 U2, which resembled similar movements to the base. The polarized microscope revealed a spread of globules surrounded by the structured colloidal network that forms a stable emulsion (Figure 6C).

In conclusion, the hydrogen bonds generated by urea promote the formation of an intercolloidal network that stabilizes the emulsion.^78−80^ However, this may not necessarily have a positive impact on the Pickering emulsion system, with the solid particles trapped on the interfacial oil globules. The movement behavior of the Pickering emulsion and the colloidal network is easily observed using a polarizing microscope, in which the presence of solid particles, either adhering to the globules (Pickering emulsion system) or forming a network in the aqueous phase, can be revealed from the different colors reflected by the crystals.

### 3.8 Antioxidant Activity and Lipid Membrane Permeation of Basil Extract in Pickering Emulsion and Colloidal Network Formulations

**Figure 7:**
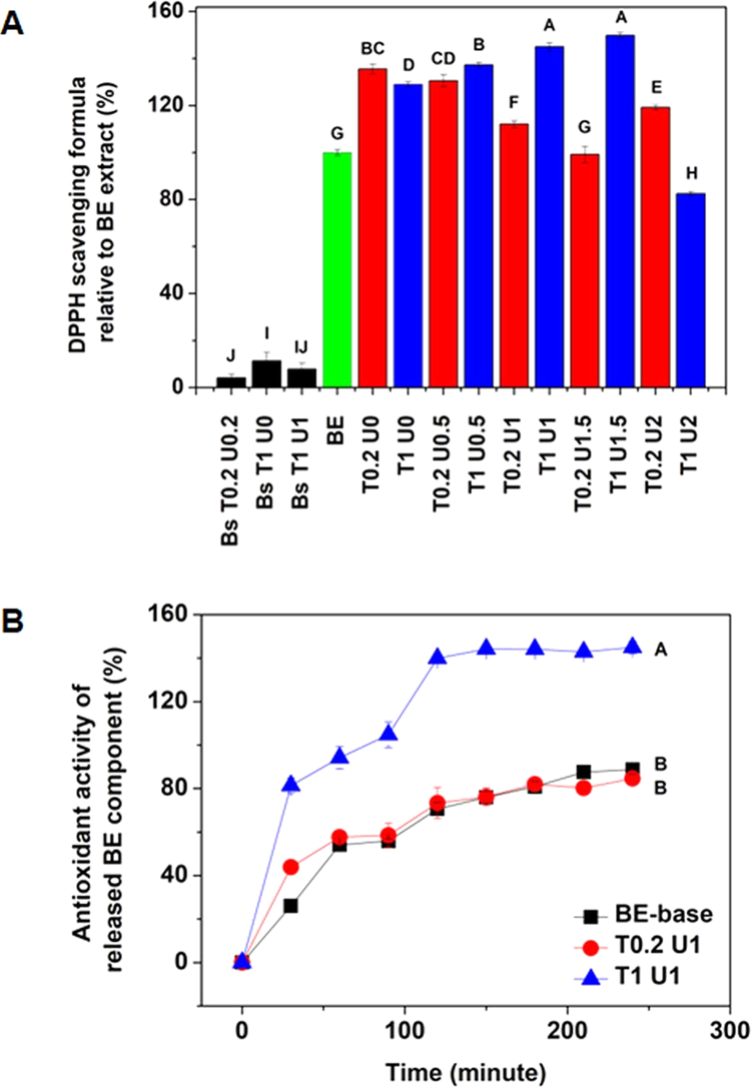
Antioxidant activity of Pickering emulsion (T0.2) and colloidal network (T1) at various concentrations of urea (A) and permeation profile through the synthetic membrane of Pickering emulsion and colloidal network (B).

Pickering emulsion provides an important alternative in the development of antiaging basil extracts, which exhibit strong antioxidant activity and potential anti-inflammatory effects.^37^ The antioxidant activity of basil extract formulated in the Pickering emulsion formulation T0.2 U0.5 and the colloidal particle network emulsion T1 U0.5 is depicted in Figure 7A. The antioxidant activity of basil extract in both systems of emulsion when added with the appropriate amount of urea showed a remarkably higher DPPH scavenging activity effect compared to the unformulated basil extract. The addition of urea had a positive effect on the system of colloidal network emulsion, with scavenging activity increasing from 129.01 ± 1.09% at the T1 U0 formula to 149.87 ± 1.17% at T1 U1.5. It is hypothesized that the urea-mediated hydrogen bonds of colloids confer the control of radical scavenging activity at high surface areas. Thus, this can explain the reduction in DPPH scavenging (82.54 ± 0.86%) in the T1 U2 formula, which had a larger particle size. This benefit is consistent with previous findings.^89,90^

A similar trend was also found in the Pickering emulsion formulation with smaller globules, which correlated with a lower amount of urea and showed higher antioxidant activity (135.54 ± 2.15% at T0.2 U0 and 130.53 ± 2.15% T0.2 U0.5). In summary, the stabilization of emulsions using antioxidants in the solid state, either adsorbed on oil globules or forming colloidal networks in the aqueous phase, provides a controlled release of antioxidant active compounds with higher activity.

This high antioxidant activity is supported by the activity of the four highest components in basil extract, as discussed in the LC-MS/MS results in Section 3.2, namely, rosmarinic acid, lariciresinol, salvigenin, and eupatorin. Rosmarinic acid and lariciresinol as polyphenols have a potent radical scavenging activity that provides a cytoprotective effect on cells, which is against the adverse effects of UV-B radiation,^91,92^ while salvigenin and eupatorin as flavonoids have anti-inflammatory activity,^93,94^ which makes this basil extract have the potential to be developed as a medical agent for ROS-induced skin diseases such as antiaging.

Antiaging formulations are required to permeate the stratum corneum to reach viable areas in the epidermis.^95,96^ It was evaluated by comparing the antioxidant activity of the permeated samples of T0.2 U1 and T1 U1 tested through synthetic lipid membranes arranged within cell diffusion.^32^ The permeation of basil extract mixed with the base formulation was used as a control. Figure 7B shows the different permeation profiles of T1 U1 compared to the other test samples. This correlates with the higher antioxidant activity of the T1 U1 formula (150%). In contrast, the T0.2 U1 formula showed an absorption pattern similar to basil extract, which was physically mixed with base (BE-base).

### 3.9 Antiaging Effect of the Formulation

#### 3.9.1 Cell Penetration and Collagen Deposition on UV-B-Irradiated Cell

**Figure 8:**
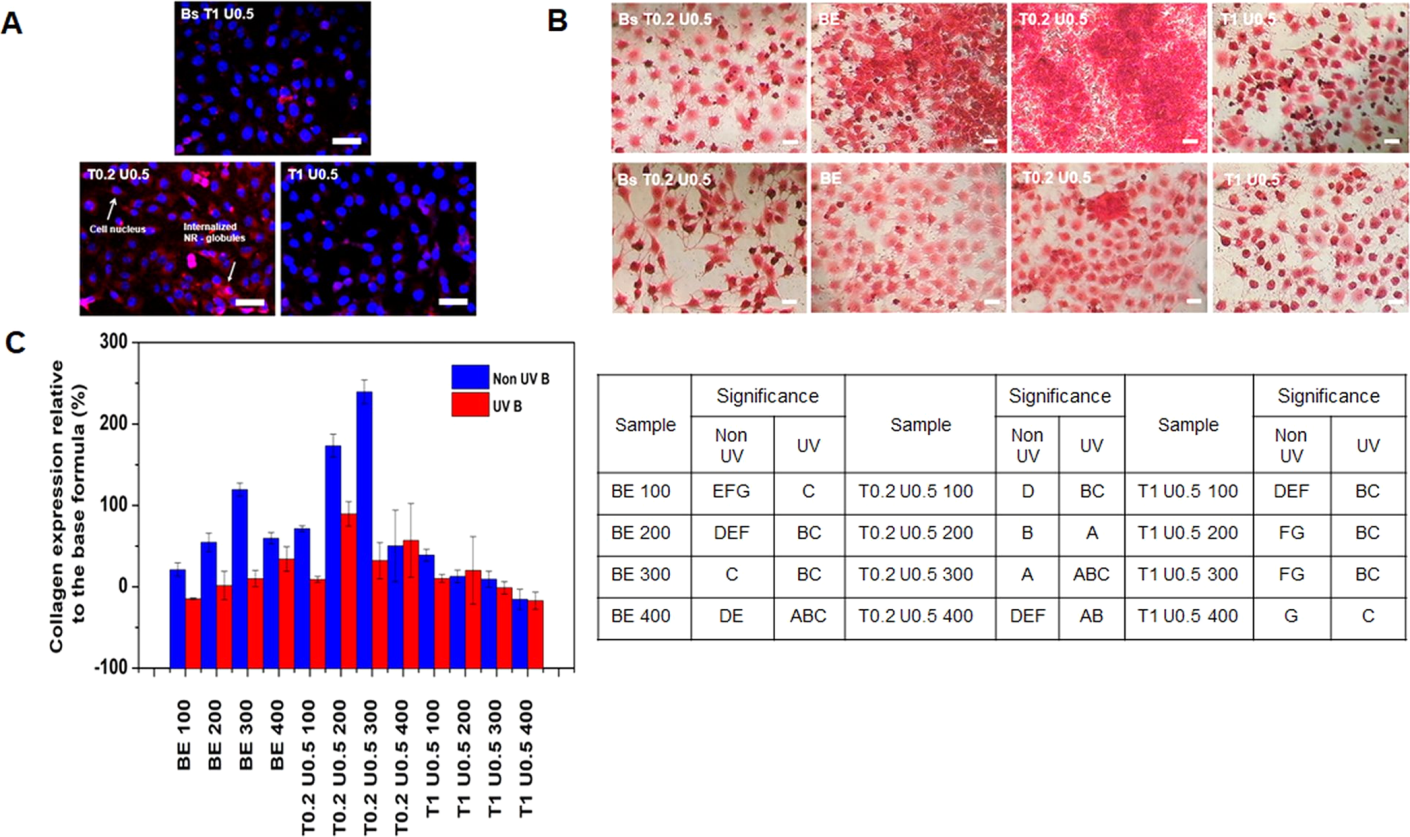
Cellular activities of O/W Pickering emulsion and colloidal network of basil extract tested on 3T3 fibroblast. (A) Cellular uptake of the base (Bs T1 U0.5), Pickering emulsion (T0.2 U0.5), and colloidal network (T1 U0.5) which were labeled using Nile red, and the penetrated samples shown as red emission surrounding cell nucleus stained with Hoechst 33342 (blue emission). Scale bar is 10 μm. (B) Qualitative measurement of collagen deposition on UV-B-irradiated cells treated with samples 1 h before irradiation and cultured for 3 days. It was shown as a red deposited color stained with picro sirius red (scale bar is 10 μm). (C) Quantitative analysis of the collagen deposition.

Cell penetration and collagen deposition on UV-B-irradiated cells were carried out using freshly prepared samples. From Figure 8A, it can be seen that under confocal microscopy, the internalized sample exhibited red fluorescence of the Nile red-labeled formula surrounding the nucleus (shown in blue with Hoechst 33342 stain). This shows that the Pickering emulsion formula T0.2 U0.5 sample can penetrate the cells and shows potential to be used as an antiaging agent to neutralize ROS generated by UV-B radiation. However, the globules were less internalized on the base of the emulsion without the basil extract of T1 U0.5 and the colloidal network emulsion T1 U0.5, which might be due to the larger globules in these samples because there is a negative correlation between the average globule size and cellular uptake.^97^

In this study, collagen deposition was tested using fibroblast as collagen producers that can be used in in vitro antiaging effects.^98^ The cells were grown overnight and treated with basil extracts, bases, and Pickering emulsions T0.2 U0.5 and T1 U0.5 1 h before UV-B irradiation. Non-UV cells were also treated using the same samples. This UV-B radiation is carried out with the aim of intervening healthy cells into damaged cells, so that in this study the effect of the sample used on healthy cells and damaged cells can be seen to evaluate the repair ability of the formula.

Using light microscope, cells without UV-B radiation and with UV-B radiation (Figure 8B) can be seen. In the Bs T0.2 U0.5 sample, the cells without UV irradiation were in normal conditions and tended to grow tightly, whereas when given UV-B radiation, the shape of the cells tends to change with the ends of the cells elongated and the cell density decreases, whereas the BE sample showed that even though there was UV-B radiation treatment, the basil extract sample could improve cell conditions, meaning that basil extract could be used effectively as an antioxidant. Likewise, in the T0.2 U0.5 sample, Pickering emulsion can effectively improve cell conditions, better than T1 U0.5 and Bs T0.2 U0.5.

Figure 8B shows collagen expression to the base formula. It can be seen that the best collagen expression was also T0.2 U0.5 in both UV-B-irradiated cells and non-UV cells. These results were significantly different from other samples (*p* < 0.05). Higher cellular uptake and collagen expression indicate the success of Pickering emulsion. This is in agreement with other studies, where Pickering emulsion enhanced cellular uptake in skin penetration.^99,100^ The structure of the oil globules was critical not only for the stability of the Pickering emulsion but also for controlling the depth of penetration and accumulation within the skin. Oils containing a solid particle stabilizer allowed the highest permeation through the skin, with linear chain oils showing the highest skin retention.

Additionally, due to its deformability, Pickering emulsions were expected to deform and pass through the cellular gaps, thereby facilitating enhanced tissue dispersion. High specific surface area favored for higher cellular affinity and led to increased cellular uptakes. The Pickering emulsion formulation T0.2 U0.5 provides better cell absorption and collagen expression than the colloidal network formulation T1 U0.5. The globule particle size in the base and colloidal network formulation T1 U0.5, which is larger than the Pickering emulsion formulation T0.2 U0.5, causes cell uptake to be difficult and the healing process to be limited. The larger particle size can reduce the adhesive contact between the cellular membrane and droplets, thereby significantly increasing the energy required to change the shape of the membrane around the droplets, leading to a decrease in cellular absorption.^97^ In the Pickering emulsion formulation T0.2 U0.5, the decrease in droplet deformability due to the decrease in droplet size can also increase cellular internalization.

The droplet particle size of the T0.2 U0.5 Pickering emulsion formulation was measured to be lower than 200 nm (113.5 nm; Table 5). This size is ideal for percutaneous permeation and cell-to-cell uptake. Thus, it can repair mitochondria and neutralize ROS that causes aging. Keratinocytes internalize via endocytosis, confined to materials about 200 nm in size. After being internalized, the adsorbed particles from phenolic contained in the Pickering emulsion formulation (5.7–22.4 nm) and colloidal network formulation (6.0–49.2 nm) are expected to be absorbed cellularly and provide effects as antioxidants and antiaging. These data provide evidence of the successful application of Pickering emulsion formulation as an antiaging induced by UV-B radiation.

Even though the T1 U0.5 formulation showed poor cellular internalization results in vitro, these results have yet to be tested in vivo. Colloidal network formulation T1 U0.5 could permeate better in vivo because the globule size of the fresh sample from colloidal emulsion T1 is also still in the range of 200 nm so that there is a tendency to be retained in the epidermis, which then, due to its deformability, will also release the solid particles, where the solid particle size in the colloidal network formulation T1 U0.5 is around 180.1 nm. Permeation of solid particles up to 198 nm can permeate well to the viable epidermal layer,^101^ so this must be tested for further in vivo activity tests planned for future research. However, the intention needs to be taken in this system not to add too much urea because the addition of urea correlates with an increase in globule size, which can decrease its cellular activity and absorption.

## 4 Conclusions

This study developed solid stabilization of the emollient formula of sunflower oil using basil extract rich in phenolic compounds. A facile method of emulsification was conducted by preconditioning extract using appropriate humectants (hexylene glycol and glycerol) and a moisturizer (urea). Surprisingly, humectants premixed with oil and saturated extract solution prior to emulsification can improve the hydrophobicity of the extract leading to in situ solidification and adhesion on the oil globules to stabilize the emulsion. In addition, the use of costabilizers (Tween 20 and urea) at optimum concentrations can produce two systems of stabilization, namely, Pickering emulsion (particles adsorbed at the oil–water interface, PE) and colloidal networks (colloidal particles that form structures in the aqueous phase, CN), both of which have antioxidant properties and different antiaging. Urea was used as a hydrogen bond donor, stabilizing colloidal particles in the aqueous phase. However, excess urea can remove particles from the oil–water interface. Therefore, it is necessary to adjust the concentration of urea so that it does not dominate one of the systems, resulting in a mixed system consisting of two stabilization systems with higher stability. In addition, the content of amphiphilic compounds in the extract also determines the success of stabilization using solid particles through their tendency to produce colloidal particles that present at the oil–water interface and the aqueous phase. Our findings provide important insights into the appropriate control of Pickering emulsification where humectants and moisturizers are usually mixed into the aqueous phase prior to emulsion formation. This research contributes to technological advances to develop green formulations of cosmetics and dermal pharmaceuticals.
